# Chemical Composition of *Artemisia annua* L. Leaves and Antioxidant Potential of Extracts as a Function of Extraction Solvents

**DOI:** 10.3390/molecules17056020

**Published:** 2012-05-21

**Authors:** Shahid Iqbal, Umer Younas, Kim Wei Chan, Muhammad Zia-Ul-Haq, Maznah Ismail

**Affiliations:** 1 Department of Chemistry, University of Sargodha, Sargodha 40100, Pakistan; 2 Laboratory of Molecular Biomedicine, Institute of Bioscience, Universiti Putra Malaysia, UPM Serdang 43400, Selangor, Malaysia; 3 Research Institute of Pharmaceutical Sciences, Department of Pharmacognosy, University of Karachi, Karachi 75270, Pakistan; 4 Department of Nutrition and Dietetics, Faculty of Medicine and Health Sciences, Universiti Putra Malaysia, UPM Serdang 43400, Selangor, Malaysia

**Keywords:** *Artemisia annua* L., chemical composition, antioxidant activity, solvent effects

## Abstract

This study was conducted to investigate the chemical and nutritional composition of *Artemisia annua* leaves in addition to determination of antioxidant potential of their extracts prepared in different solvents. Chemical composition was determined by quantifying fat, protein, carbohydrate, fiber, tocopherol, phytate, and tannin contents. Extraction of *A. annua* leaves, for antioxidant potential evaluation, was carried out using five solvents of different polarities, *i.e.*, hexane, chloroform, ethyl acetate, methanol and water. Antioxidant potential was evaluated by estimating total phenolic (TPC), flavonoid (TFC) contents, ferric reducing antioxidant power (FRAP), Trolox equivalent antioxidant capacity (TEAC), DPPH radical scavenging activity and lipid peroxidation. Efficiency of different solvents was compared for the yield of antioxidant extracts from leaf samples and a clear variation was observed. The highest TPC, TFC, TEAC, DPPH radical scavenging and lowest lipid peroxidation were observed in MeOH extracts, whereas aqueous extract exhibited high ferric reducing antioxidant power; suggesting MeOH to be the most favorable extractant.

## 1. Introduction

Reactive oxygen species (ROS) and free radicals like superoxide, hydroxyl, peroxyl, hydroperoxyl and alkoxyl are produced in the human body as a result of normal metabolism [1]. On the basis of epidemiological studies, nowadays, there is ample evidence for the involvement of ROS in a number of chronic (atherosclerosis, diabetes, and cancers) [2], cardiovascular and neurodegenerative diseases; by causing oxidative damages to DNA, proteins, lipids and other small cellular molecules [3]. Sometimes, excessive generation of ROS takes place in human body due to many exogenous factors resulting in oxidative stress and because the built-in antioxidant system may not be efficient enough to counteract overpopulation of these radicals. To overcome this state of stress, antioxidant supplements from external sources are required [4]. Synthetic antioxidants like butylated hydroxy-anisole (BHA) and butylated hydroxytoluene (BHT) have been in use for a long time as food additives, but recent reports on their involvement in a number of diseases have restricted their use and BHA/BHT have eventually been banned in many developed countries [5]. These concerns have prompted food scientists, researchers and health professionals to search for alternative sources of antioxidants based on natural origin, which may be safer, more effective and economical, preferably from plant materials based on indigenous resources. As a result of these investigations, a number of plant materials and plant constituents were explored as promising sources of antioxidants. But there still exists a need to explore more viable sources, development of new media and techniques for extraction with improved yields coupled with methodology for accurate determination of their antioxidant activities.

Antioxidant compounds, present in plants, are of diverse structure and their activity in different model systems and extractability is strongly dependent on their chemical structure, so different extraction media, *i.e.*, solvent systems, may provide varying yields of extracts with selective recovery of antioxidants; depending upon the structure of antioxidant compounds present in the plant material under investigation [6]. Interference in antioxidant determining assays may occur due to a number of factors like non-antioxidant components and the polarity of extracting solvents. Therefore, different solvent systems must be employed for extraction followed by comparison of yield and antioxidant potential of extracts from any plant material. Extracting solvents are also reported to affect the efficiency of antioxidant determination assays in the following sequence: ORAC > ABTS > DPPH > FRAP. The mechanistic details suggest that polar solvents may mainly affect to only those methods, which involve H-atom donation (e.g., ORAC and ABTS), rather than the assays which involve electron transfer, e.g., FRAP [7].

Among the potential medicinal plants of antioxidant properties, *A. annua* exhibits a number of promising characteristics. It is a single stem herb of 2–3 m height, with a dynamic growth ability. Many bioactive compounds have been reported from this plant [8]. Among these, artemisinin is a well proven antimalarial drug [9], and semi-synthetic derivatives of artemisinin have reported to display good pharmacological performances against opportunistic pathogens which cause pneumonia in AIDS and among other patients having a feeble immune system [8]. This plant is also known to have antipyretic, antiparasitic, antiulcerogenic and anti-inflammatory properties [10].

Being an inhabitant of moderate atmospheres, *Artemisia* is mostly found in the northern hemisphere at mid to high latitude in arid and semi-arid environmental landscapes. Central Asia hosts a number of species of *Artemisia* L. [11] and out of them, 25 are found in Pakistan and most of them are present in the northern regions, *i.e.*, the Himalaya, Hindu Kush and Karakorum mountain ranges in the Abbottabad, Chitral, Gilgit, Kashmir, Rawalpindi, Skardu and Swat districts [12]. Locally, this plant is known by the name of *Afsantin* or *Afsantin jari* and the whole plant is used against malaria; especially the leaves are used for cough, common cold, diarrhea and fevers [11].

Some preliminary analysis on the antioxidant properties of *Artemisia annua* leave extracts has been presented. But to the best of our knowledge, no detailed investigation on its antioxidant activity, via different assays (based on different principles of determination) and function of different extraction solvents in better recovery of antioxidant components, has been presented so far. Therefore, this study was planned to get a clear understanding about the impact of solvent polarity on extraction of antioxidant compounds from *A. annua* leaves coupled with determination of antioxidant activity through multiple assays. These investigations will play a key role in recovery of bioactive components, which are reported to be acting against certain diseases associated with oxidative stress, which may eventually lead to development of new functional foods and nutraceutical products.

## 2. Results and Discussion

### 2.1. Chemical Composition

The chemical composition of *Artemisia annua* leaves is summarized in Table 1. Percentage of ash, carbohydrate, fat, fiber, moisture and protein is calculated on a dry weight basis. The lesser proportion of nutrients (fat and protein), and higher amount of anti-nutrients (phytate and total tannin), as compared to previously reported composition of *A. annua* leaves [8]; indicate the weaker nutritive action of these samples. However, the ash content suggests a high amount of inorganic minerals in *A. annua* leaves. Carbohydrate and fat proportions may contribute towards their health promoting efficiency.

Fibre is known as an anti-nutrient, as it has the potential to obstruct the utilization of minerals. In addition to this, it assists phytate, tannins and oxalates besides hampering the mineral absorption by binding them with itself [13]. High amounts of crude fibre in *A. annua* leaves, as compared to traditional vegetables, may restrict their more frequent consumption [14]. However, higher protein content than leafy vegetables [15] signifies a good antioxidant ability of the tested leaf samples [16].

Phytates are also labeled as anti-nutrients due to their protein binding ability that may reduce protein solubility [17] as well as bioavailability of minerals (Ca and Zn) to the consumer [18]. Higher concentration of phytates, in tested leaf samples than documented for leaves of *Solanum nigrum* and *Leonotis leonorus* [19] may increase their anti-nutritive behavior. Tannins are polyphenols, which form insoluble complexes with iron through *O*-dihydroxyl groups and result in reduction of iron absorption in the gastrointestinal tract [13], while the antioxidant activity of tannins is also well known [20]. The amount of tannins calculated in *A. annua* leaves may not have any considerable contribution towards their functional attributes.

The dire need of vitamins for good health of mammals can never be denied. Tocopherols (vitamin E) are lipophilic antioxidants and exist in four isomeric forms, *i.e.*, α, β, γ and δ tocopherols; α-tocopherol being the major one. These vitamins are continuously synthesized by plants and are stored in green tissues, *i.e.*, leaves [21]. The value of total tocopherols estimated in *A. annua* leaves suggests their promising potential for functional foods and nutraceuticals.

### 2.2. Total Phenolic Content (TPC)

The antioxidant properties of phenolic compounds are well known; they are potent chelators of redox-active metal ions and they can inactivate free radical chain reactions by hindering the conversion of hydroperoxides to reactive oxyradicals [22]. Significantly different amounts of phenolics were found in the various extracts of *A. annua* leaves, ranging from 90.12 ± 2.78 (hexane) to 134.50 ± 4.37 (MeOH).

**Figure 1:**
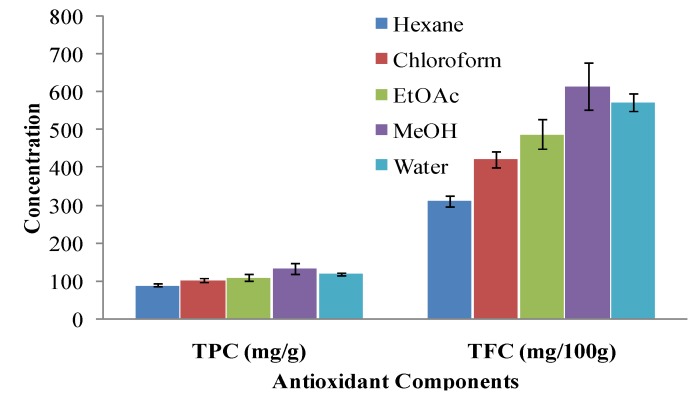
Contents of phenolics and flavonoids in various solvent extracts of *Artimisia annua* leaves.

In many studies, yield of phenolics extraction has shown a strong correlation with the polarity of the solvent used; high polarity solvents being the best for extraction [23]. Therefore, solvents including acetone, dimethylformamide, ethanol, ethyl acetate, methanol and propanal have been tested for the extraction of phenolics [24]. The efficiency of different solvents for the extraction of phenolics was found to be in the order: MeOH > water > EtOAc > chloroform > hexane (Figure 1).

It was observed that with the use of high polarity solvents, recovery of TPC was also improved and the highest concentration of phenolics was found in MeOH extracts; confirming the ability of methanol to solubilize a larger fraction of the phenolic components present in *A. annua* leaves. Water showed comparable potential as that of methanol regarding extraction of TPC. Arabshahi-Delouee *et al.* reported similar results for mulberry (*Morus indica* L.) leaves [25]. All these observations suggest that most of the phenolic compounds are highly polar and are extractable in polar solvents.

### 2.3. Total Flavonoid Content (TFC)

The free radical scavenging activity of flavonoids by making complexes with metal ions is well proven [26]. The trend of flavonoids extraction, among various solvents, was same as observed in case of phenolics (Figure 1), *i.e.*, flavonoids were more effectively extracted in a polar solvent, *i.e.*, methanol (614.98 ± 18.14) than any other solvent used for extraction. These results are in agreement with Spingo *et al.* [27], who suggested that polar solvents are the best extracting media for flavonoids, which may be due to an increase in polarity of flavonoids upon conjugation through glycosides with hydroxyl groups that enhances their solubility in polar solvents [28].

### 2.4. Ferric Reducing Antioxidant Power (FRAP) Assay

Ferric reducing antioxidant power assay has been used successfully for the estimation of antioxidant capacity of number of plant materials including *Olea europaea* L. [29] and *Zanthoxylum piperitum* leaves [30]. This assay is based on single electron transfer and interprets the ferric reduction capacity of samples [31]. An oxidant probe, *i.e.*, ferric ion, accepts an electron from antioxidant species in the sample and converts itself to ferrous state, which on chelating with a chromogenic ligand (tripyridyltriazine, TPTZ) gives a colored complex having a maximum absorbance at 593 nm, that is proportional to the ferric reducing antioxidant power of the sample [32].

**Figure 2:**
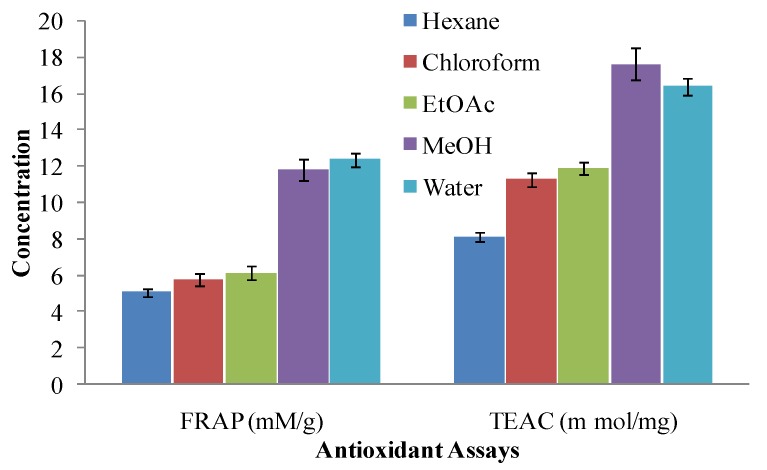
Ferric reducing antioxidant power and trolox equvilant antioxidant capacity of various solvent extracts of *Artimisia annua* leaves.

Influence of solvent polarity on the FRAP assay was reported recently [33], which was also conspicuous in the present work. The FRAP values for hexane, chloroform and EtOAc extracts were found to be mutually comparable but clearly different than those for polar solvent, *i.e.*, MeOH extract (11.82 ± 1.12). Maximum FRAP value was 12.37 ± 1.09 but it is also comparable to MeOH extract, when a highly polar solvent, *i.e.*, water was added for extraction (Figure 2). Beyond the fact that FRAP assay is inadequate for the determination of hydrophilic (water soluble) antioxidants [34], the reduction of maximum ferric ions in aqueous extracts confirmed the extraction efficiency of polar solvents.

### 2.5. Trolox Equivalent Antioxidant Capacity (TEAC) Assay

Trolox equivalent antioxidant capacity assay portrays the ability of antioxidants to scavenge long life radicals like ABTS^•**+**^ [33]. These radicals generate colored solutions having a maximum absorbance at 746 nm. Antioxidant species present in the sample reduce the color intensity of the solution by neutralizing free radicals, which helps to estimate an accurate TEAC value [35]. This method is widely preferred due to its efficiency to evaluate antioxidant capacity of food items and biological matrices [36].

The Trolox equivalent antioxidant capacity of *A. annua* leaves extracts was found to be in the range of 8.12 ± 0.21 to 17.59 ± 0.71 for hexane and methanol respectively (Figure 2). The magnitude of reaction of *A. annua* leaves extracts, prepared in different solvents, against ABTS^•**+**^ radical was found in the order as: MeOH > water > EtOAc > chloroform > hexane. TEAC of polar solvents were found significantly higher than those for non-polar solvents. Hexane, chloroform, and EtOAc extracts of leaves offered comparable resistance against this assay but addition of polar solvents (MeOH and water) for extraction purposes changed the whole scenario and high values of TEAC were observed. It is reported that TEAC assay may evaluate true antioxidant potential of sample extracted in any medium; as its reagents are soluble in aqueous as well as organic solvents. Moreover, this assay can evaluate the potential of lipohilic (fat soluble) and hydrophilic (water soluble) antioxidants [34]. Keeping this in mind, it may be concluded that polar solvents possess better potential to extract the maximum antioxidants from *A. annua* leaves.

### 2.6. 2,2-Diphenyl-2-picrylhydrazyl Hydrate (DPPH) Radical Scavenging Assay

This method has generally been used for the antioxidant activity evaluation of biological samples including leaves. DPPH (2,2-diphenyl-2-picrylhydrazyl hydrate) is a nitrogen centered radical having maximum absorbance at 517 nm, which gets converted to 1,1,diphenyl-2-picryl hydrazine on reacting with hydrogen donating species [6], *i.e.*, antioxidants present in the sample, especially polyphenols [37]. This hydrogen donation ability leads towards formation of stable complex of free radicals, resulting in termination of lipid peroxidation [38].

**Figure 3:**
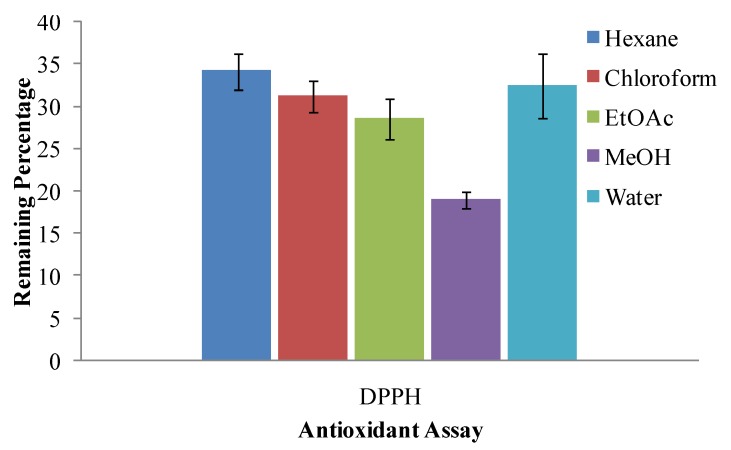
DPPH radical scavenging potential of various solvent extracts of *Artimisia annua* leaves.

The DPPH radical scavenging potential of the MeOH extract of *A. annua* leaves was found to significantly higher than all other extracts. Efficiency of extracts against DPPH assay was of same fashion as that observed for TEAC (Figure 3). The extracts prepared in less polar solvents (hexane, chloroform and EtOAc) acted against DPPH radical with comparable power but MeOH extracts came up with the highest scavenging potential, which indicated the presence of maximum antioxidant constituents in MeOH extracts and mark methanol as potential solvent for the extraction of antioxidants from *A. annua* leaves.

### 2.7. Lipid Peroxidation

The unsaturated sites in the structures of fatty acids are easily attacked by free radicals, which promote the formation and regulation of lipid radicals. In this process, double bonds of lipids undergo rearrangement, result in destruction of lipids and produce breakdown products such as malondialdehyde. The estimation of malondialdehyde in a plant extract helps to evaluate its protection level against lipid peroxidation, *i.e.*, the resistance of antioxidants present in sample against hazardous effects of free radicals on unsaturated fatty acids to minimize the production of malondialdehyde [39].

**Figure 4:**
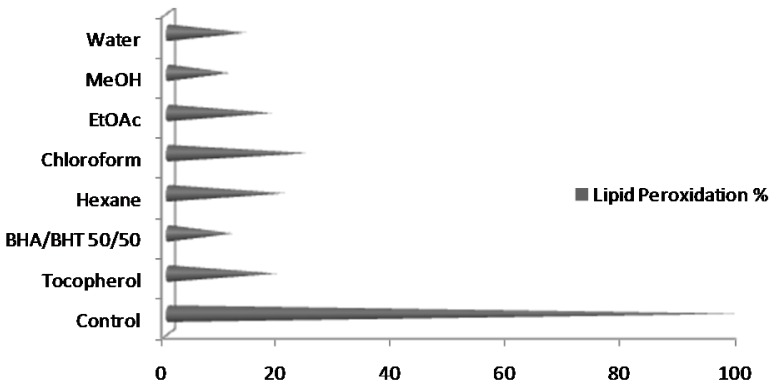
Lipid peroxidation in extracts of *Artimisia annua* leaves prepared in different solvents.

In case of *A. annua* leaves extracts, highest lipid peroxidation (24.24 ± 1.04%) was observed in chloroform extract, and the lowest (10.72 ± 0.07) in MeOH extract. The comparison of lipid peroxidation among *A. annua* leave extracts prepared in different solvents is presented in Figure 4.

A mixture of two synthetic antioxidants (BHA/BHT, 50/50) and one natural antioxidant (tocopherol) is used for comparison. Lipid peroxidation in MeOH extract was lesser than standard synthetic as well as natural antioxidants.

### 2.8. Statistical Analysis

Correlation analysis (Table 2) exhibited positive and strong correlation of TPC with TFC (r = 0.972), FRAP (r = 0.866), TEAC (r = 0.971) and DPPH (r = 0.825). The findings suggest strong involvement of phenolics in the antioxidant activity of *A. annua* leaves, while high negative correlation of TPC was observed with lipid peroxidation, which proved that high TPC may cause a reduction in lipid peroxidation. Total flavonoid content also exhibited strong correlation with FRAP (r = 0.887) and TEAC (r = 0.980), while its correlation was moderate with DPPH, which confirms their contribution towards antioxidant actions.

## 3. Experimental

### 3.1. Chemicals and Reagents

The reagents and solvents used (hexane, chloroform, ethyl acetate and methanol) were of analytical grade and procured from Fisher Scientific, Leicestershire, UK; while Folin-Ciocalteu reagent, 2,4,6-tripyridyl-s-triazine (TPTZ), 2,2′-azino-bis(3-ethylbenzthiazoline-6-sulphonic acid) (ABTS), 1,1-diphenyl-2-picrylhydrazyl (DPPH), α-tocopherol, thiobarbituric acid and gallic acid were purchased from Sigma-Aldrich and phosphate buffer from R&M Chemicals, Bristol, UK.

### 3.2. Collection of Samples and Extraction of Antioxidants

Fresh samples of *A. annua* leaves were collected from hilly peripheral areas of Islamabad region, Pakistan. The leaves were chopped after washing with running water, dried in oven at 40 °C for 2 days followed by grinding to powder [30]. Ground *A. annua* leaves were extracted individually with five different solvents, *i.e.*, hexane, chloroform, ethyl acetate, methanol and water sequentially using a Soxhlet apparatus. Extraction time was kept 6 h for each solvent [6]. All the extracts were filtered through a 0.45 mm nylon membrane filter, dried under reduced pressure at 45 °C using a rotary evaporator and stored at −20 °C prior to further analyses [40].

### 3.3. Chemical Composition

The samples were analyzed for chemical composition (protein, fat, carbohydrates, ash, fibre and moisture) following AOAC protocol [41]. Tannins were determined following a method of Makker and Goodchild [42], phytate contents were estimated using a method of Wheeler and Ferrel [43] and tocopherols were quantified following a procedure described by Barros *et al.* [44].

### 3.4. Estimation of Total Phenolic Contents (TPC)

The extracts of *A. annua* leaves, prepared in different solvents, were subjected to determination of total phenolic content (TPC) using Folin-Ciocalteu (FC) reagent assay [40]. Briefly, extract (200 μL) was mixed with freshly prepared and diluted (1:10) FC reagent (750 μL) and sodium carbonate solution (2 mL, 7.5%). The reaction mixture was diluted by adding deionized water to make the total volume up to 7 mL, placed in dark for 2 h and the reaction was allowed to complete. Absorbance of resulting solution was measured at 765 nm and TPC was calculated using gallic acid as standard and results were presented as gallic acid equivalents (mg/g). These proceedings were executed thrice for each sample and results were averaged.

### 3.5. Estimation of Total Flavonoid Contents (TFC)

The measurement of TFC was done following a colorimetric assay [40]. One milliliter of extract was added to a 10 mL volumetric flask already having deionized H_2_O (4 mL) followed by the addition of 5% NaNO_2_ solution (0.3 mL). After 5 min, a similar amount of AlCl_3_ solution (10%) was added and at the 6th min, 1 M NaOH solution (2 mL) was added. Flask contents were diluted by adding deionized H_2_O (2.4 mL) and were thoroughly mixed. Absorbance of reaction mixture was measured at 510 nm. TFC of the samples were determined as epicatechin equivalents (mg/100 g). In the same way, TFC of all the extracts were determined thrice and results were averaged.

### 3.6. Estimation of Antioxidant Activity

#### 3.6.1. Ferric Reducing Antioxidant Power (FRAP) Assay

The FRAP assay reported by Benzie and Strain [45] was used to evaluate ferric reducing antioxidant power of *A. annua* leaves extracts prepared in different solvents. Briefly, FRAP reagent was prepared by mixing acetate buffer (300 mM, pH 3.6), 2,4,6-tripyridyl-s-triazine (TPTZ) solution (10 mM TPTZ in 40 mM/HCl) and FeCl_3_.6H_2_O (20 nM) in the ratio of 10:1:1 and 12 mL deionized water at 37 °C. In order to perform FRAP assay, FRAP reagent (1.8 mL), Milli-Q water (180 µL) and sample (60 µL) were added to the same test tube, placed at 37 °C for 4 min. The absorbance of reaction mixture was measured at 593 nm using FRAP working solution as blank. Ferric reducing antioxidant power of all the extracts was determined thrice in comparison with FeSO_4_•7H_2_O standard curve.

#### 3.6.2. Trolox Equivalent Antioxidant Capacity (TEAC) Assay

Trolox equivalent antioxidant activity of extracts was assessed by estimating the ABTS**^•+^** radical cation scavenging potential of *A. annua* leaves extracts. For TEAC evaluation, the method reported by Iqbal *et al.* [46] was used. The preparation of free radical cation was done by oxidizing 5 mmol/L solutions of ABTS with MnO_2_ for half an hour. On the other hand, each extract was diluted in 5 mmol/L phosphate buffered saline (PBS, pH 7.4) to an absorbance of about 0.700 at 734 nm. The diluted extract (2.5 mL) was added to radical solution (7.0 mL) and after 10 min the absorbance was noted using PBS as blank. In the same way, absorbance of all the extracts was measured and antioxidant activity was calculated using Trolox as standard and results were expressed as Trolox equivalent mmol/mg of dried leaves.

#### 3.6.3. DPPH**^•^** Scavenging Assay

Estimation of DPPH radical scavenging activity of various solvent extracts of *A. annua* leaves was done according to method of Iqbal *et al.* [47]. Leaf extract (1.0 mL) was taken and freshly prepared DPPH solution (5.0 mL) was added. The decrease in absorbance was measured at 515 nm at different intervals, *i.e.*, 1, 2, 5, 7 and 10 min till the absorbance reduced to 50%. The standard calibration curve was used to calculate the remaining concentration (%) of DPPH. The remaining DPPH at 5th min was used to compare the scavenging potential of different *A. annua* extracts.

#### 3.6.4. Lipid Peroxidation

Lipid peroxidation in terms of malondialdehyde (MDA) contents was evaluated following a method of Heath and Packer [48]. Briefly, dried extract (0.5 mg) was homogenized with 1% trichloroacetic acid (TCA, 5 mL) and centrifuged at 15,000 *g* for 10 min. Supernatant (0.5 mL) was mixed with a solution (2 mL) prepared by mixing 20% TCA containing 0.5% thiobarbituric acid (TBA). The resulting mixture was heated at 96 °C for 30 min, followed by cooling at 0 °C and centrifugation at 10,000 g for 10 min at 4 °C. Absorbance was measured at 532 and 600 nm using extinction coefficient of 155 mM^−1^cm^−1^.

### 3.7. Statistical Analysis

All the experiments were conducted thrice, results were averaged and data are presented as mean ± standard deviation in Figure 1, Figure 2, Figure 3 and Figure 4 and Table 1. Correlation among different antioxidant components and antioxidant assays was studied by pearson’s correlation coefficient (Table 2).

## 4. Conclusions

Various solvents having different polarities were employed for the extraction of antioxidant components present in *A. annua* leaves, and estimation of antioxidant activity was done in those extracts using different assays. Our study confirms the difference in extraction efficiency of the various solvents, which suggests that the solvent effect should be taken into account while addressing the antioxidant potential of any sample. From this study, a suggestion regarding use of methanol for the extraction can be made, if *A. annua* leaves are to be used as functional food or medicine. The evaluation of antioxidant competency of *A. annua* leaves by means of different assays recommends the use of these leaves as nutraceuticals.

## Figures and Tables

**Table 1:** Chemical composition of *Artemisia annua* leaves.

Sr. No	Contents	Amount (% dry weight basis)
1	Ash	7.5
2	Carbohydrate	8.3
3	Fat	6.07
4	Fibre	14.2
5	Moisture	11.4
6	Protein	24.37 mg/100 g
7	Phytate	140.4
8	Total Tannins	0.61
9	Tocopherol	2.74

**Table 2:** Correlation coefficient between TPC, TFC, Lipid peroxidation, FRAP, TEAC and DPPH assays.

	TPC	TFC	FRAP	TEAC	DPPH	L.P
**TPC**	1					
**TFC**	0.972	1				
**FRAP**	0.866	0.887	1			
**TEAC**	0.971	0.980	0.949	1		
**DPPH**	0.825	0.698	0.493	0.671	1	
**L.P**	−0.832	−0.824	−0.884	−0.845	−0.643	1

## References

[1] Teow C.C., Truong V.D., McFeeters R.F., Thompson R.L., Pecota K.V., Yencho G.C. (2007). Antioxidant activities, phenolic and [beta]-carotene contents of sweet potato genotypes with varying flesh colours. Food Chem..

[2] Pihlanto A., Akkanen S., Korhonen H.J. (2008). ACE-inhibitory and antioxidant properties of potato (*Solanum tuberosum*). Food Chem..

[3] Lai F., Wen Q., Li L., Wu H., Li X. (2010). Antioxidant activities of water-soluble polysaccharide extracted from mung bean (*Vigna radiata* L.) hull with ultrasonic assisted treatment. Carbohydr. Polym..

[4] Anagnostopoulou M.A., Kefalas P., Papageorgiou V.P., Assimopoulou A.N., Boskou D. (2006). Radical scavenging activity of various extracts and fractions of sweet orange peel (*Citrus sinensis*). Food Chem..

[5] Yingming P., Ying L., Hengshan W., Min L. (2004). Antioxidant activities of several Chinese medicine herbs. Food Chem..

[6] Jayaprakasha G.K., Girennavar B., Patil B.S. (2008). Antioxidant capacity of pummelo and navel oranges: Extraction efficiency of solvents in sequence. LWT Food Sci. Technol..

[7] Çelik S.E., Özyürek M., Güçlü K., Apak R. (2010). Solvent effects on the antioxidant capacity of lipophilic and hydrophilic antioxidants measured by CUPRAC, ABTS/persulphate and FRAP methods. Talanta.

[8] Brisibe E.A., Umoren U.E., Brisibe F., Magalhäes P.M., Ferreira J.F.S., Luthria D., Wu X., Prior R.L. (2009). Nutritional characterisation and antioxidant capacity of different tissues of *Artemisia annua* L.. Food Chem..

[9] Teresa C., Rossana P., Maria P.A., Paride P., Francesco P.F., Luciano V., Pinarosa A. (2012). Phytochemical analysis of a herbal tea from *Artemisia annua* L.. J. Pharm. Biomed. Anal..

[10] Sanja C., Milka M., Danijiela V., Adisa P. (2012). Chemical composition and antioxidant and antimicrobial activity of essential oil of *Artemisia annual* L. from Bosnia. Ind. Crops Prod..

[11] Hayat M.Q., Khan M.A., Ashraf M., Jabeen S. (2009). Ethnobotany of the genus *Artemisia* L. (Asteraceae) in Pakistan. Ethnobotany Res. App..

[12] Abdul M., Ibrar A., Waheed A., Muhammad A., Rizwana Q., Izhar H., Bushra M. (2010). Survey of artemisinin production by diverse Artemisia species in northern Pakistan. Malar. J..

[13] Brinch-Pedersen H., Borg S., Tauris B., Holm P.B. (2007). Molecular genetic approaches to increasing mineral availability and vitamin content of cereals. J. Cereal Sci..

[14] Odhav B., Beekrum S., Akula U., Baijnath H. (2007). Preliminary assessment of nutritional value of traditional leafy vegetables in KwaZulu-Natal, South Africa. J. Food Compost. Anal..

[15] Gupta S., Jyothi Lakshmi A., Manjunath M., Prakash J. (2005). Analysis of nutrient and antinutrient content of underutilized green leafy vegetables. LWT Food Sci. Technol..

[16] Maisuthisakul P., Pasuk S., Ritthiruangdej P. (2008). Relationship between antioxidant properties and chemical composition of some Thai plants. J. Food Compost. Anal..

[17] Krishnan H.B., Natarajan S.S. (2009). A rapid method for depletion of Rubisco from soybean (*Glycine max*) leaf for proteomic analysis of lower abundance proteins. Phytochemistry.

[18] Wallace P., Marfo E., Plahar W. (1998). Nutritional quality and antinutritional composition of four non-conventional leafy vegetables. Food Chem..

[19] Jimoh F., Adedapo A., Afolayan A. (2010). Comparison of the nutritional value and biological activities of the acetone, methanol and water extracts of the leaves of *Solanum nigrum* and *Leonotis leonorus*. Food Chem. Toxicol..

[20] Sathaye S., Bagul Y., Gupta S., Kaur H., Redkar R. (2011). Hepatoprotective effects of aqueous leaf extract and crude isolates of *Murraya koenigii* against *in vitro* ethanol-induced hepatotoxicity model. Exp. Toxicol. Pathol..

[21] Barros L., Carvalho A.M., Ferreira I.C.F.R. (2010). Leaves, flowers, immature fruits and leafy flowered stems of *Malva sylvestris*: A comparative study of the nutraceutical potential and composition. Food Chem. Toxicol..

[22] Sahreen S., Khan M.R., Khan R.A. (2010). Evaluation of antioxidant activities of various solvent extracts of *Carissa opaca* fruits. Food Chem..

[23] López A., Rico M., Rivero A., de Tangil M.S. (2011). The effects of solvents on the phenolic contents and antioxidant activity of *Stypocaulon scoparium* algae extracts. Food Chem..

[24] Alothman M., Bhat R., Karim A. (2009). Antioxidant capacity and phenolic content of selected tropical fruits from Malaysia, extracted with different solvents. Food Chem..

[25] Arabshahi-Delouee S., Urooj A. (2007). Antioxidant properties of various solvent extracts of mulberry (*Morus indica* L.) leaves. Food Chem..

[26] Jung C.H., Seog H.M., Choi I.W., Park M.W., Cho H.Y. (2006). Antioxidant properties of various solvent extracts from wild ginseng leaves. LWT Food Sci. Technol..

[27] Spigno G., Tramelli L., de Faveri D.M. (2007). Effects of extraction time, temperature and solvent on concentration and antioxidant activity of grape marc phenolics. J. Food Eng..

[28] Mohsen S.M., Ammar A.S.M. (2009). Total phenolic contents and antioxidant activity of corn tassel extracts. Food Chem..

[29] Hayes J., Allen P., Brunton N., O’Grady M., Kerry J. (2011). Phenolic composition and *in vitro* antioxidant capacity of four commercial phytochemical products: Olive leaf extract (*Olea europaea* L.), lutein, sesamol and ellagic acid. Food Chem..

[30] Jeong C.H., Kwak J.H., Kim J.H., Choi G.N., Kim D.O., Heo H.J. (2010). Neuronal cell protective and antioxidant effects of phenolics obtained from *Zanthoxylum piperitum* leaf using *in vitro* model system. Food Chem..

[31] Wootton-Beard P.C., Moran A., Ryan L. (2011). Stability of the total antioxidant capacity and total polyphenol content of 23 commercially available vegetable juices before and after *in vitro* digestion measured by FRAP, DPPH, ABTS and Folin Ciocalteu methods. Food Res. Int..

[32] Berker K.I., Guclu K., Tor I., Apak R. (2007). Comparative evaluation of Fe (III) reducing power-based antioxidant capacity assays in the presence of phenanthroline, batho-phenanthroline, tripyridyltriazine (FRAP), and ferricyanide reagents. Talanta.

[33] Perez-Jimenez J., Saura-Calixto F. (2006). Effect of solvent and certain food constituents on different antioxidant capacity assays. Food Res. Int..

[34] Tyug T.S., Prasad K.N., Ismail A. (2010). Antioxidant capacity, phenolics and isoflavones in soybean by-products. Food Chem..

[35] Santas J., Carbo R., Gordon M., Almajano M. (2008). Comparison of the antioxidant activity of two Spanish onion varieties. Food Chem..

[36] van den Berg R., Haenen G.R.M.M., van den Berg H., Bast A. (1999). Applicability of an improved Trolox equivalent antioxidant capacity (TEAC) assay for evaluation of antioxidant capacity measurements of mixtures. Food Chem..

[37] Turkmen N., Sari F., Velioglu Y.S. (2006). Effects of extraction solvents on concentration and antioxidant activity of black and black mate tea polyphenols determined by ferrous tartrate and Folin-Ciocalteu methods. Food Chem..

[38] Akowuah G., Ismail Z., Norhayati I., Sadikun A. (2005). The effects of different extraction solvents of varying polarities on polyphenols of *Orthosiphon stamineus* and evaluation of the free radical-scavenging activity. Food Chem..

[39] Michielin E.M.Z., Wiese L.P.L., Ferreira E.A., Pedrosa R.C., Ferreira S.R.S. (2011). Radical-scavenging activity of extracts from *Cordia verbenacea* DC obtained by different methods. J. Supercrit. Fluids.

[40] Iqbal S., Bhanger M. (2006). Effect of season and production location on antioxidant activity of *Moringa oleifera* leaves grown in Pakistan. J. Food Compost. Anal..

[41] Association of Official Analytical Chemists (AOAC) (1995). Official Methods of Analysis.

[42] Makkar H.P.S., Goodchild A.V. (1996). Quantification of Tannins: A Laboratory Manual.

[43] Wheeler E., Ferrel R. (1971). A method for phytic acid determination in wheat and wheat fractions. Cereal Chem..

[44] Barros L., Heleno S.A., Carvalho A.M., Ferreira I.C.F.R. (2009). Systematic evaluation of the antioxidant potential of different parts of *Foeniculum vulgare* Mill. from Portugal. Food Chem. Toxicol..

[45] Benzie I.F.F., Strain J. (1996). The ferric reducing ability of plasma (FRAP) as a measure of “antioxidant power”: The FRAP assay. Anal. Biochem..

[46] Iqbal S., Bhanger M., Anwar F. (2007). Antioxidant properties and components of bran extracts from selected wheat varieties commercially available in Pakistan. LWT Food Sci. Technol..

[47] Iqbal S., Bhanger M., Anwar F. (2005). Antioxidant properties and components of some commercially available varieties of rice bran in Pakistan. Food Chem..

[48] Heath R.L., Packer L. (1968). Photoperoxidation in isolated chloroplasts: I. Kinetics and stoichiometry of fatty acid peroxidation. Arch. Biochem. Biophys..

